# Ruptured visceral artery aneurysms in a patient of neurofibromatosis type 1 (NF-1) successfully treated by endovascular treatment

**DOI:** 10.1186/s40792-020-0791-6

**Published:** 2020-01-13

**Authors:** Naoko Fukushima, Hiroaki Aoki, Shinsuke Takenaga, Kazuhiko Morikawa, Masaichi Ogawa, Katsuhiko Yanaga

**Affiliations:** 1 Department of Surgery, Katsushika Medical Center, The Jikei University, School of Medicine, 6-41-2 Aoto, Katsushikaku, Tokyo, 125-8506 Japan; 2 Department of Radiology, Katsushika Medical Center, The Jikei University, School of Medicine, 6-41-2 Aoto, Katsushikaku, Tokyo, 125-8506 Japan; 3 Department of Surgery, The Jikei University, School of Medicine, 3-19-18 Nishishinbashi, Minatoku, Tokyo, 105-8471 Japan

**Keywords:** Visceral artery aneurysms, Neurofibromatosis type 1, Endovascular treatment

## Abstract

**Background:**

Neurofibromatosis type 1 (NF-1) is an autosomal dominant disease and arteriovenous abnormalities are a well-recognized complication. There are several case reports of ruptured aneurysms; however, among them, reports of superior pancreaticoduodenal artery (PDA) and superior mesenteric artery (SMA) aneurysms are rare. We experienced the case of ruptured PDA and SMA aneurysms in a patient of neurofibromatosis type I successfully treated by endovascular treatment.

**Case presentation:**

A 55-year-old woman with NF-1 came to our hospital with abdominal pain and vomiting. Enhanced abdominal computed tomography revealed a hematoma in the retroperitoneum and an aneurysm in the head of the pancreas. Angiography was performed, and a ruptured aneurysm was suspected the periphery of the PDA, and we embolized it using coils. However, on postoperative day 2, the hemoglobin level decreased, and a branch of the SMA was ruptured. She underwent embolization using coils again and discharged on postoperative day 27 without any further hemorrhage.

**Conclusions:**

To our knowledge, this is the first successfully treated case of ruptured SMA and PDA aneurysms in a patient with NF-1.

## Background

Neurofibromatosis type 1 (NF-1) is an autosomal dominant disorder affecting one in 3000 people which is characterized by café au lait spots and various features in the bones, eyes, and nervous system. Vascular abnormalities such as arterial stenosis, aneurysms, arterial compression, and arteriovenous abnormalities have also been known [1, 2], of which involvement of renal arteries aneurysms is the most common. However, aneurysms of intracranial, cranial, vertebral, subclavian, intercostal, and visceral arteries are also reported, and spontaneous rupture may occur with fragile vessel walls [3]. We herein report a case of ruptured visceral artery aneurysms in a patient with NF-1 successfully treated by endovascular treatment and review the literature.

## Case presentation

**Fig. 1:**
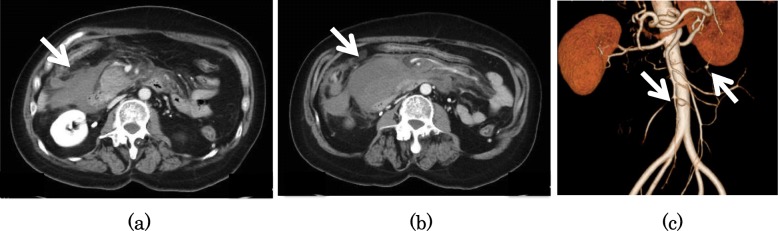
Enhanced abdominal computed tomography showed a hematoma in the retroperitoneum (**a**), a lesion suggestive of an aneurysm in the head of the pancreas (**b**), and multiple aneurysms arising from the jejunal artery (**c**)

**Fig. 2:**
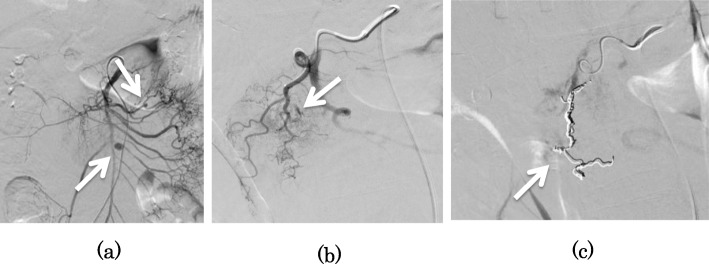
Angiography revealed two jejunal aneurysms without extravasation (**a**). PDA angiogram showed a slightly enlarged branch artery with extravasation (**b**). Coil embolization was performed from the distal side to the proximal side of the enlarged artery (**c**)

**Fig. 3:**
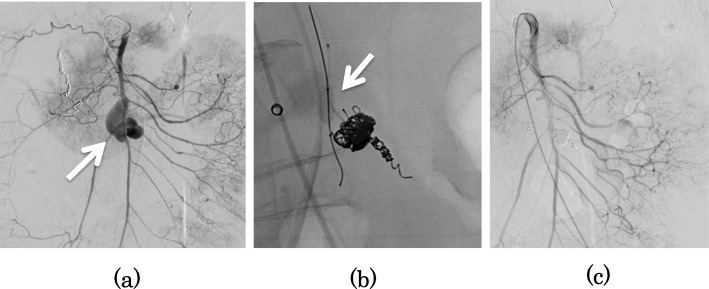
Angiography revealed a jejunal aneurysm with massive extravasation (**a**). Embolization was performed using detachable coils in the distal side of the aneurysm. Subsequently, we embolized the aneurysm by filling the same coils. Finally, coils were deployed into the proximal side of the aneurysm during balloon occlusion of SMA to avoid coil migration (**b**). SMA angiogram after embolization, jejunal blood flow was well maintained (**c**)

A 55-year-old woman was brought to our hospital by ambulance for acute abdominal pain and vomiting. She had a past medical history of NF-I and schizophrenia. She had no history of hypertension, diabetes, and hyperlipemia. She had multiple café au lait spots on her body. Her blood pressure was 149/103 mmHg, heart rate was 105 beats/min, and body temperature was 36.9 °C. Physical examination revealed mild tenderness in the upper abdomen. Laboratory findings revealed signs of inflammatory reaction (white blood cell count 18,800/μL and C-reactive protein level 0.23 mg/dL) and anemia (hemoglobin 11.7 g/dL). Enhanced abdominal computed tomography showed a hematoma in the retroperitoneum, multiple aneurysms arising from the jejunal artery, and a lesion suggestive of an aneurysm in the head of the pancreas (Fig. 1). Hence, we suspected rupture of visceral artery aneurysms and performed emergency angiography, which revealed an aneurysm arising from the jejunal artery (Fig. 2a) and a lesion with extravasation of blood at the periphery of the superior pancreaticoduodenal artery (PDA) (Fig. 2b). We judged the periphery of PDA as a responsible lesion and embolized from the distal side to the proximal side of the aneurysm using several pushable coils (C-stopper coil, Piolax Medical Devices Inc., Kanagawa, Japan) (Fig. 2c). However, on postoperative day 2, the hemoglobin level decreased to 6.3 g/dL, and hence emergency angiography was performed again, which revealed a lesion with extravasation from a branch of superior mesenteric artery (SMA) which was not detected at the first time (Fig. 3a). We deployed several detachable coils (Target, Stryker, Fremont, CA, USA) into the distal side of the ruptured jejunal aneurysm and then embolized the aneurysm by filling the same detachable coils. To avoid proximal migration of coils into the main trunk of SMA, the same detachable coils were deployed into the proximal side of the aneurysm during the occlusion SMA of the orifice of the jejunal branch using a microballoon catheter (Attendant SP, Terumo Clinical Supply, Gifu, Japan) (Fig. 3b). After embolization, we confirmed that the jejunal blood flow was maintained on SMA angiogram (Fig. 3c). On the next day, hemostasis was confirmed by repeat angiography. However, she developed delayed gastric emptying due to compression of hematoma, which slowly disappeared, and she was discharged on postoperative day 27. Since there are still some aneurysms, we will follow the aneurysms by computed tomography once a year.

## Discussion

NF-1 is a multisystem genetic disorder caused by the mutation of a gene on chromosome 17. Common symptoms include cutaneous findings, most notably café au lait spots and neurofibromas, skeletal dysplasia, tumors of the central and peripheral nervous system, and vision disorders [4]. NF-1 is also associated with vascular abnormalities, which may be complicated by spontaneous rupture [5]. The causes of rupture are reported as follows: (1) Neurofibromatous infiltrates directly into the tunica media, and the blood vessel wall becomes weak. (2) Neurofibromatous compress the vasa vasorum of the large artery tissue and the blood vessel wall becomes weak. (3) Tunica media becomes weak because of the smooth muscle proliferation in tunica intima and the elastic membrane becomes weak [1, 6].

Most lesions become evident by age 50 years [7]. Most patients with NF-1 vasculopathy are reported to be asymptomatic, but the patients with ruptured artery aneurysms have an acute presentation with hemodynamic instability and loss of consciousness with localized pain and swelling which could be fatal [3, 5]. In the current case, hemodynamic was stable, but she had a sudden abdominal pain.

Stanley et al. reported that the frequency of visceral artery aneurysm occurrence is 60% in the splenic, 20% in the hepatic, 5.5% in the superior mesenteric, 4% in the celiac, 4% in the gastric or gastroepiploic, 3% in the intestinal and colon, 2% in the pancreaticoduodenal or pancreatic, 1.5% in the gastroduodenal, and less than 1% in the inferior mesenteric arteries [8]. As in the current case, the aneurysms of PDA and SMA are rare, and only one case of ruptured PDA aneurysm and two cases of ruptured SMA aneurysm have been reported in patients with NF-1 [2, 9, 10], which are listed in Table 1.

**Table 1:** Three cases of the rupture of SMA and PDA aneurysm

Author	Year reported	Age	Sex	Bleeding source	Treatment	Outcome
Mendonça et al.	2010	31	F	SMA	Embolization	Alive
Huffman et al.	1996	44	–	SMA	Operation	Alive
Serleth et al.	1998	20	M	PDA	Operation	Alive

Treatment for ruptured aneurysm includes surgery and endovascular treatment, but there is no consensus. It depends on the type, patient’s age, and the location of the lesion. The advantage of endovascular treatment is that it is less invasive, excellent in organ preservation, and rapid recovery [11, 12]. Recently, there have been some cases for which hemorrhage was successfully terminated by endovascular treatment, which seems to be increasingly performed. David et al. reported that endovascular treatment even in hemodynamically unstable patients at all ages appears to be a safe approach [5]. However, there are problems such as difficulty in the procedure, possibility of intestinal necrosis, and report of vascular damage from coils and catheters. To avoid vascular injury due to the fragility of the vessel wall, it is necessary to select appropriately sized coils that match the diameter of the blood vessel or aneurysm. Additionally, a softer coil should be selected as we used in the current case. In particular, it is important to be careful with the trouble of the insertion site in NF-1 because of the weakening of the vessel wall [10, 11]. Open surgery may be required in some cases. In the current case, we chose endovascular treatment because of its minimum invasiveness, and the ruptured aneurysms were successfully treated without intestinal ischemia. Although the follow-up period is not decided, the visceral aneurysms in patients with NF-1 are more likely to rupture, and it is necessary to perform enhanced CT at least 1 year. In the current case, if the jejunal aneurysms are getting bigger, the treatment, such as the coil embolization, will be required again.

## Conclusions

We experienced the case of ruptured PDA and SMA aneurysms in a patient of neurofibromatosis type I successfully treated by endovascular treatment. To our knowledge, this is the first successfully treated case of ruptured PDA and SMA aneurysms in a patient with NF-1.

## Abbreviations

NF-1: Neurofibromatosis type 1
PDA: Superior pancreaticoduodenal artery
SMA: Superior mesenteric artery
